# Passive Immunisation against RHDV2 Induces Protection against Disease but Not Infection

**DOI:** 10.3390/vaccines9101197

**Published:** 2021-10-18

**Authors:** Robyn N. Hall, Tegan King, Tiffany W. O’Connor, Andrew J. Read, Sylvia Vrankovic, Melissa Piper, Tanja Strive

**Affiliations:** 1 Health & Biosecurity, Commonwealth Scientific and Industrial Research Organisation, Acton, ACT 2601, Australia; tegan.king4891@gmail.com (T.K.); Tanja.Strive@csiro.au (T.S.); 2 Centre for Invasive Species Solutions, Bruce, ACT 2617, Australia; 3 Elizabeth Macarthur Agricultural Institute, NSW Department of Primary Industries, Menangle, NSW 2568, Australiaandrew.j.read@dpi.nsw.gov.au (A.J.R.); sylvia.vrankovic@dpi.nsw.gov.au (S.V.); 4 Agriculture & Food, Commonwealth Scientific and Industrial Research Organisation, Acton, ACT 2601, Australia; Melissa.Piper@csiro.au

**Keywords:** RHDV2, lagovirus, rabbit calicivirus, maternal antibody, passive immunity, biocontrol

## Abstract

Rabbit haemorrhagic disease virus 2 (RHDV2) is a lagovirus in the family *Caliciviridae*. The closely related Rabbit haemorrhagic disease virus (RHDV, termed RHDV1 throughout this manuscript for clarity) has been used extensively as a biocontrol agent in Australia since the mid-1990s to manage wild rabbit populations, a major economic and environmental pest species. Releasing RHDV1 into populations with a high proportion of rabbits less than 8–10 weeks of age leads to non-lethal infection in many of these young animals, with subsequent seroconversion and long-term immunity against reinfection. In contrast, RHDV2 causes lethal disease even in young rabbits, potentially offering substantial benefits for rabbit management programs over RHDV1. However, it is not clear how acquired resistance from maternal antibodies may influence immunity after RHDV2 infection. In this study, we assessed serological responses after RHDV2 challenge in young rabbits of three different ages (5-, 7-, or 9-weeks-old) that were passively immunised with either high- (titre of 2560 by RHDV IgG ELISA; 2.41 mg/mL total protein) or low- (titre of 160–640 by RHDV IgG ELISA; 1.41 mg/mL total protein) dose RHDV2 IgG to simulate maternal antibodies. All rabbits treated with a high dose and 75% of those treated with a low dose of RHDV2 IgG survived virus challenge. Surviving animals developed robust lagovirus-specific IgA, IgM, and IgG responses within 10 days post infection. These findings demonstrate that the protection against RHDV2 conferred by passive immunisation is not sterilising. Correspondingly, this suggests that the presence of maternal antibodies in wild rabbit populations may impede the effectiveness of RHDV2 as a biocontrol.

## 1. Introduction

Two lagoviruses are known to be pathogenic in European rabbits (*Oryctolagus cuniculus*)—RHDV1 (genotype GI.1) and RHDV2 (genotype GI.2) [1]. Both cause an acute fulminant hepatitis leading to disseminated intravascular coagulation, multi-organ failure, and death in susceptible rabbits within 24–72 h post infection. However, unlike RHDV1, RHDV2 can also infect several *Lepus* (hares and jackrabbits) and *Sylvilagus* (cottontails) species [2,3,4,5,6,7,8]. RHDV2 also causes disease in young rabbits, which show an age-dependent innate resistance to lethal disease caused by RHDV1 despite being permissive to infection [9]. RHDV2 is antigenically distinct from RHDV1, overcoming both infection-induced and vaccinal immunity [10,11].

Wild European rabbits are one of Australia’s most significant agricultural and environmental vertebrate pest species [12,13]. Lagoviruses, because of their high virulence, species specificity, and capacity to transmit at a landscape scale, are used as biocontrol agents to help manage overabundant wild rabbit populations. Currently, two RHDV1 variants, v351 (genotype GI.1c) and RHDVa-K5 (genotype GI.1a), are approved for use in Australia. A significant limitation of these variants is that they should not be used in populations with a high proportion of young rabbits, which is rarely practical or achievable. While young animals may become infected, intrinsic age-dependent resistance to lethal disease with these variants leads to many surviving and acquiring life-long immunity against reinfection [14,15]. The mechanism underlying this innate resistance to RHDV1 in young rabbits is not fully understood, but it is known to be independent of maternal antibody status [16], can be abrogated by immunosuppression [17], and correlates with constitutively heightened innate immune responses, particularly those associated with major histocompatibility class II molecules, natural killer cells, macrophages, and cholangiocytes [18,19].

This innate resistance to disease is further complicated by the presence of benign, typically enterotropic, rabbits caliciviruses (RCV) that induce partial immunological cross-protection against virulent variants [20,21,22,23,24,25,26,27], and by maternal antibodies in populations where RHDV1 is endemic. Maternal antibodies are known to attenuate the immune response to both natural infection and vaccination for pathogens such as canine parvovirus, leading to vaccine failure [28]. When considering lagoviruses as biocontrol agents, the presence of maternal antibodies could theoretically result in two potential outcomes: (1) ‘sterilising’ immunity (i.e., protection from both disease and infection), resulting in neutralisation of challenge virus without seroconversion and no protection against re-infection, or (2) ‘attenuating immunity’ (i.e., protection from disease but not infection), leading to seroconversion and long-term protection against subsequent re-infection. These contrasting outcomes dramatically influence the use of lagoviruses as biocontrol agents. Robinson et al. [16] confirmed that RHDV1 maternal antibodies were protective against lethal disease. The survival of young rabbits after intramuscular (IM) challenge (1500 rabbit infectious doses (RID) as determined from in vivo titrations) was dependent on both kitten age and the antibody titre of the doe but not of the kitten, which in many cases were too low to be reliably detected in serological assays. While some kittens seroconverted after virus challenge (30/41), notably those from does with low antibody titres, 27% remained seronegative [16]. Parkes et al. [29] additionally showed that 9-week-old (wo) rabbits born to seropositive does survived ultra-low dose RHDV1 challenge (1.5 RID and 2.5 RID; oral inoculation), most without seroconverting (11/12), and remained fully susceptible when rechallenged eight weeks later. This may suggest that maternal antibody-mediated protection is less significant than innate age-dependent resistance for driving population immunity against RHDV1 in wild rabbits.

In rabbits, maternal antibodies are acquired from the doe via active transport across the placenta in the final days of pregnancy [30,31]. Unlike ruminant species, rabbits do not acquire antibodies via colostrum, and while antibodies can be acquired through lactation, this route plays only a minor role [31]. Maternal antibodies in rabbits are exclusively of the IgG isotype and decline with age and, correspondingly, with increasing bodyweight [16,22,32]. For non-pathogenic lagoviruses, maternal antibodies wane between 4 and 7 weeks of age, depending on the titre of the doe [22]. Similarly, maternal antibodies against RHDV2 decline between 28 and 58 days (4 and 8.3 weeks) of age [31]. In contrast, maternal antibodies against RHDV1 persist for up to 12 weeks in some animals, although typically they wane by eight weeks of age [32]. In Australian wild rabbits, maternal antibodies against RHDV1 were detectable in most animals of <500 g bodyweight, half of the animals around 800 g bodyweight, and in very few individuals >1 kg [32].

Unlike RHDV1, RHDV2 can lethally infect rabbits of all ages, making it an attractive prospect as an additional biocontrol agent for potential use year-round, irrespective of the breeding season and changes in population age profiles. However, the impact of maternal antibodies on subsequent viral challenge has not yet been investigated for RHDV2 in this context. In this study, we aimed to determine whether maternal immunity to RHDV2 was sterilising or attenuating by challenging passively immunised rabbit kittens (to simulate maternal antibodies) and monitoring for survival and seroconversion. If passive immunisation against RHDV2 were to induce sterilising immunity, kittens would become susceptible once maternal antibodies have waned and could then be targeted with repeat applications. In contrast, should maternal immunity be attenuating, care would need to be taken during the breeding season to minimise potential adverse long-term outcomes for rabbit biocontrol programs.

## 2. Materials and Methods

### 2.1. Production and Purification of RHDV2 IgG

All work was conducted in accordance with the ‘Australian code for the care and use of animals for scientific purposes’ and was approved by the institutional animal ethics committee (CWLA-AEC #2018-06 and #2016-01). Animals were confirmed to be seronegative to known lagoviruses prior to inclusion in experiments by the RHDV IgG isotype ELISA (which is highly cross-reactive between all lagoviruses and does not distinguish between RHDV1 and RHDV2) [22,33] and the RCV blocking ELISA [34].

Three adult female New Zealand white (NZW) rabbits were used for the production of RHDV2 hyperimmune serum. These rabbits were vaccinated subcutaneously with 1 mL of an experimental formalin-inactivated RHDV2 vaccine produced as previously described [35], and were orally challenged six days post vaccination (dpv) with 1 × 10^8^ capsid gene copies of the RHDV2 (genotype GI.1bP-GI.2) isolate BlMt-1 (passage 1) (GenBank accession number KT280060) [36,37]. The rabbits were humanely killed 22 days post challenge (28 dpv) by intravenous (IV) barbiturate injection, following IM sedation with 5 mg/kg xylazine and 30 mg/kg ketamine. Sera were collected and stored at −20 °C.

Sera were pooled, clarified at 10,000× *g* for 30 min at 4 °C, and filtered sequentially through 0.8 µM mixed cellulose ester filters and 0.45 µM polyvinylidene fluoride (PVDF) filters. Filtered serum was diluted in PBS (between 1/10 to 1/40, dependent on batch) so that the final pH was between 7 and 8. IgG was purified from diluted serum by fast protein liquid chromatography (FPLC) using 5 mL of CaptureSelect IgG-Fc (rabbit) affinity resin (Thermo Fisher Scientific, Waltham, MA, USA) packed into a Cytiva XK 16/40 column (GE Healthcare). FPLC was performed at room temperature using an AKTA Purifier system with the following conditions: 5 column volumes (CV) equilibration with PBS pH 7.2–7.4, direct sampling loading (50–150 mL aliquots), 10 CV wash with PBS pH 7.2–7.4, 7–10 CV elution with 0.1 M glycine pH 3.0, and 5 CV re-equilibration with PBS pH 7.2–7.4. Elution fractions were neutralised with 0.1 volume tris-HCl pH 8.0. Elution fractions were pooled and dialysed against PBS overnight at 4 °C and were filter-sterilised prior to in vivo use using a 0.2 µM PVDF syringe filter. A portion of the final RHDV2 IgG preparation was concentrated 5-fold using Amicon Ultra-15 centrifugal filter units with 100 KDa nominal molecular weight limit (Merck Millipore, Burlington, MA, USA). The neat (low-dose) and concentrated (high-dose) RHDV2 preparations were aliquoted into single-use vials and stored at −80 °C. Quality of the RHDV2 IgG preparations were assessed using the Qubit protein assay (Thermo Fisher Scientific), RHDV IgG isotype ELISA [22,33], RHDV2 IgA and IgM isotype ELISAs [35], and reducing SDS-PAGE with Coomassie stain (Supplementary Figure S1).

### 2.2. Pharmacokinetics of Rabbit Polyclonal Serum

To assess the pharmacokinetics of rabbit polyclonal serum, we treated 12 wo NZW rabbits with 0.1 mL/kg, 0.3 mL/kg, or 0.5 mL/kg of hyperimmune RHDV2 polyclonal serum by either IM or IV injection (*n* = 1 per group). The hyperimmune serum was produced as part of a previous study [35]. The serum was filtered sequentially through 0.8 µM, 0.45 µM, and 0.2 µM syringe filters to ensure sterility prior to injection. For IV injections, animals were first sedated with 5 mg/kg xylazine and 30 mg/kg ketamine administered IM. To monitor RHDV IgG levels over time, serum (50–250 μL) was collected prior to treatment (baseline), 5 min post injection (D0), and then twice daily for 4.5 days (D0.5, D1, D1.5, D2, D2.5, D3, D3.5, D4, D4.5).

### 2.3. Passive Immunisation Trials

**Figure 1:**
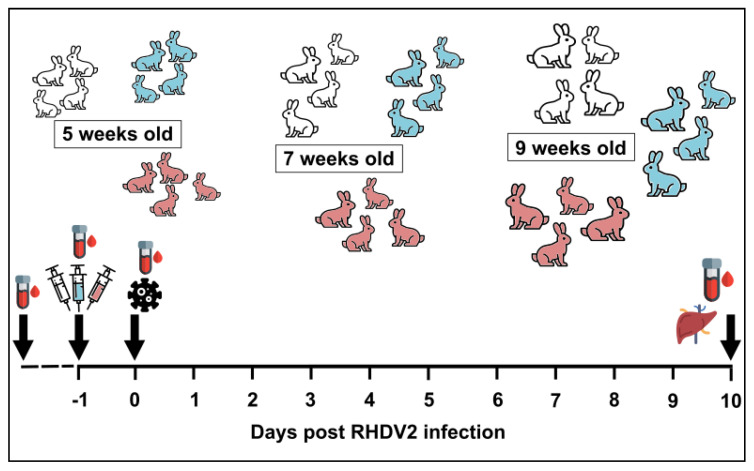
Experimental design. Rabbits aged 5-, 7-, or 9-weeks old were passively immunised with either a high (pink) or low (blue) dose of RHDV2 IgG or PBS (white) by intramuscular injection in groups of 4 animals per treatment group. They were challenged 24 h later with 50 RID_50_ of RHDV2 and were monitored for the development of terminal rabbit haemorrhagic disease. Sera were collected for serological analyses at baseline, at the time of passive immunisation (day −1), at the time of infection (day 0), and at the end of the trial (day 10). Liver samples were collected on day 10 for viral RNA quantification using RT-qPCR.

Rabbits (NZW) aged 5-, 7-, or 9-wo were passively immunised IM with 0.5 mL/kg of either ‘low dose’ (titre of 160–640 by RHDV IgG ELISA) or ‘high dose’ (titre of 2560 by RHDV IgG ELISA) purified RHDV2 IgG (four animals per age group per dose); control animals were included with each age group and received PBS (Figure 1). Sample sizes were determined in consultation with a biostatistician based on a power calculation with assumptions of <5% survival probability in infected kittens with no antibody protection, a 20% survival probability with low levels of maternal antibodies, a 50% survival probability with moderate levels of maternal antibodies, and an 80% survival probability with high levels of maternal antibodies. The IM route was selected based on our findings from the *‘Pharmacokinetics of rabbit polyclonal serum*’ experiments described above. The RHDV IgG ELISA is highly cross-reactive between all lagoviruses and does not distinguish between RHDV1 and RHDV2. Twenty-four hours after passive immunisation, rabbits were challenged orally with 50 times the 50% rabbit infectious dose (RID_50_ as determined by in vivo titration; equivalent to 3 × 10^5^–6 × 10^6^ capsid copies [37]) of a genotype GI.1bP-GI.2 RHDV2, MEN-1 (GenBank accession number MW467791) [9]. This variant is homologous to that used for the preparation of the RHDV2 IgG, described above, sharing 98.3% nucleotide identity across the genome. The release of RHDV1 as a rabbit biocontrol agent in Australia is regulated as an oral bait, which utilises the natural oral route of infection. The 50 RID_50_ dose was estimated to be what a rabbit may reasonably consume on carrot or oat bait when the virus is reconstituted as directed. Rabbits were housed individually in a climate-controlled, insect-proof facility with *ad libitum* access to food (Ol’ Jack Rabbit premium pellets, Laucke Mills) and water, and were provided with environmental enrichment.

Rabbits were monitored twice daily and those with terminal rabbit haemorrhagic disease (RHD), indicated by a rectal temperature less than 38 °C with concurrent weight loss and lethargy, were humanely killed by IV barbiturate overdose after sedation with IM xylazine (5 mg/kg) or medetomidine (130 µg/kg), and ketamine (30 mg/kg). To continuously monitor activity levels and external body temperature profiles, rabbits were fitted with collars comprising a FitBark2 activity monitor (FitBark Inc., Kansas City, MO, USA) and SubCue-Mini temperature datalogger (Canadian Analytical Technologies Inc., Calgary, Canada), as previously described [9]. Trials were terminated once humane endpoints were reached or at 10 days post infection (dpi), allowing sufficient time for seroconversion to occur [35].

Serum was collected at the time of passive immunisation, at the time of infection, and at post-mortem to monitor for seroconversion. Liver samples were collected to look for evidence of viral replication after challenge. All samples were stored at −80 °C.

### 2.4. RNA Extraction and RT-qPCR

RNA was extracted from 20 mg of liver using the SimplyRNA Tissue kit and Maxwell RSC16 instrument (Promega, Madison, WI, USA), as per the manufacturer’s instructions. Absolute quantification of viral RNA was performed in duplicate using a universal lagovirus SYBR Green-based RT-qPCR assay targeting a conserved region of VP60 and calibrated against a standard curve prepared using RNA in vitro transcripts, as previously described [37].

### 2.5. Serological Analyses

A series of lagovirus-specific ELISAs were used to investigate the immune responses to virus challenge after passive immunisation. These assays have been described previously [33,35]. The characteristics of the different assays are presented in Table 1; importantly, the RHDV IgG ELISA is broadly cross-reactive and detects all lagoviruses, including RHDV2, with high sensitivity. Results are reported either as the corrected optical density (OD) (the ratio of the OD of the sample to the OD of the negative control sera measured at 492 nm for sera diluted 1/40) or as a titre. For the RHDV2 IgM, RHDV2 IgA, and RHDV IgG assays the titres were calculated as the reciprocal of the highest serum dilution with an OD reading at least two times that of the negative control serum at the same dilution. For analytical purposes, titres less than 1/40 are reported as “0”.

### 2.6. Data Analysis

SubCue-Mini temperature logger data and Fitbark2 activity data were downloaded from their respective software applications and analysed in Microsoft Excel and R version 4.0.5 [38]. Plots were generated using ggplot2 [39] and ggpubr [40]. Survival analyses were done using survminer [41]. Other R packages used during this study for data manipulation and statistical analyses include readxl [42], cowplot [43], pracma [44], and those in the tidyverse [45].

## 3. Results

### 3.1. Production and Purification of RHDV2 IgG

RHDV2 IgG was purified from hyperimmune rabbit serum using FPLC, dialysed against PBS, and filter sterilised. An aliquot of this was concentrated to generate a ‘high dose’ preparation. FPLC was selected as the purification method of choice after trialing several alternative approaches. Briefly, purification of rabbit IgG using ammonium sulphate precipitation (at 35%, 40%, 45%, or 50%) or the Nab Protein A Plus spin kit (Thermo Scientific) led to increased IgM and IgA co-purification and reduced purity of the IgG preparation compared to FPLC purification (methods and data available on request). The total protein concentration of the purified IgG (by FPLC) was 2.41 mg/mL for the ‘high dose’ concentrated preparation and 1.41 mg/mL for the ‘low dose’ preparation.

While we observed some co-purification of IgM and IgA isotypes, enrichment for IgG reduced the RHDV2 IgM titres from 40,960 in the unpurified sample to 2560 in the ‘high dose’ preparation and to 40 in the ‘low dose’ preparation. Similarly, the RHDV2 IgA titres reduced from 10,240 in the unpurified sample to 2560 in the ‘high dose’ preparation and to 640 in the ‘low dose’ preparation. The RHDV IgG titre of the purified RHDV2 IgG was 2560 for the ‘high dose’ preparation and between 160 to 640 (accounting for interassay variation) for the ‘low dose’ preparation. Note that equivalent titres do not imply that the isotypes are present in the same absolute concentrations [46]. Reducing SDS-PAGE showed that the RHDV2 IgG was highly purified compared to the unpurified hyperimmune serum (Supplementary Figure S1).

### 3.2. Pharmacokinetics of Rabbit Polyclonal Serum

**Figure 2:**
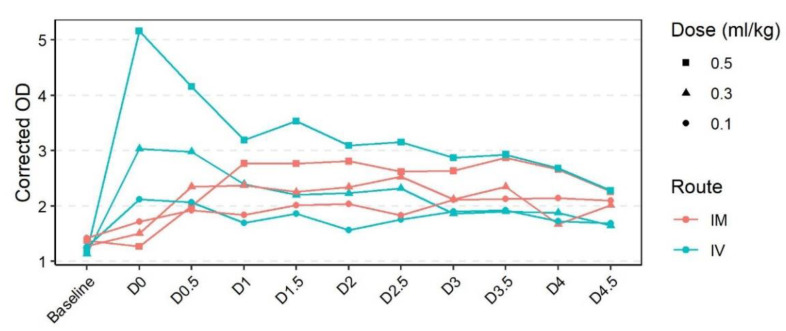
RHDV IgG ELISA reactivity after administration of RHDV2 hyperimmune polyclonal serum. Rabbits were given RHDV2 polyclonal serum by either intravenous (IV) or intramuscular (IM) injection. RHDV IgG reactivity was measured in serum collected prior to treatment (baseline), 5 min post injection (D0), and then twice daily for 4.5 days (D0.5, D1, D1.5, D2, D2.5, D3, D3.5, D4, D4.5). Results are expressed as the corrected optical density (OD) (the ratio of the OD of the sample to that of the negative control sera).

Prior to assessing the effects of passive immunization on virus challenge, we first assessed the pharmacokinetics of different doses of RHDV2 IgG and compared the IM and IV routes of administration. A clear dose-dependent increase in RHDV IgG ELISA reactivity was observed 5 min post IV RHDV2 polyclonal serum administration, but not after IM administration (Figure 2). This initial peak in reactivity immediately began to decline, while in animals treated IM, the reactivity gradually increased over time. By one day, post treatment reactivity was comparable between rabbits treated by the IV or IM routes and remained stable until at least 4.5 days post treatment for both IM and IV treated animals and at all doses tested. Thus, for subsequent experiments, rabbits were passively immunised by the IM route 24 h prior to virus challenge.

### 3.3. Effect of Passive Immunisation on Disease and Infection

**Figure 3:**
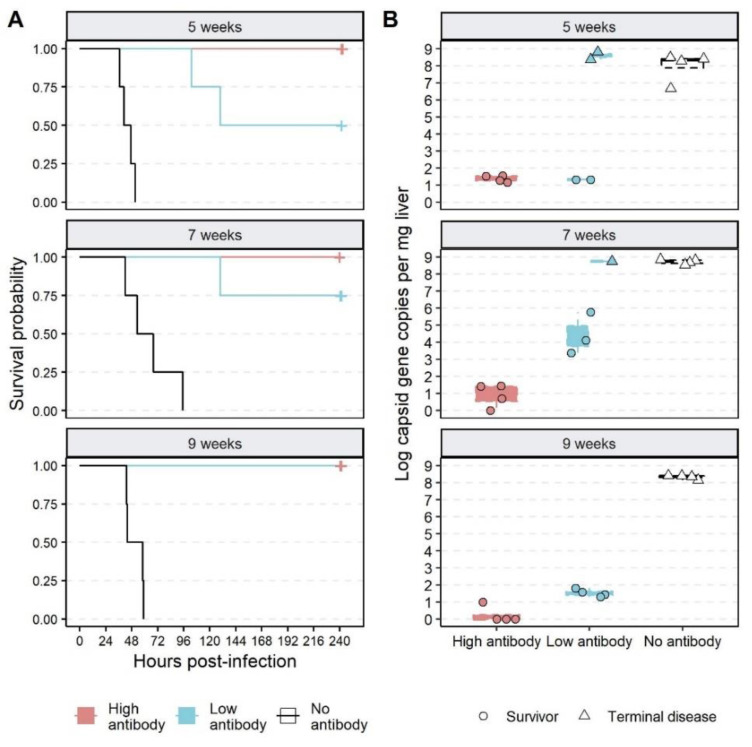
Survival curves and viral RNA loads of passively immunised rabbits after RHDV2 infection. Rabbits aged 5-, 7-, or 9-weeks old were passively immunised with either a high (pink) or low (blue) dose of RHDV2 IgG or PBS (white) by intramuscular injection in groups of four animals per treatment group. They were challenged 24 h later with 50 RID_50_ of RHDV2 and were monitored for the development of terminal rabbit haemorrhagic disease. (**A**) Survival time was obtained from continuous temperature monitors. Survival analysis was performed using the survminer package. (**B**) Total RNA was extracted from post-mortem liver samples and viral RNA was quantified by SYBR-based RT-qPCR. Individual data points and summary boxplots are shown, coloured by dose of RHDV2 IgG. Triangles represent rabbits that developed terminal disease while dots represent animals that survived infection (liver samples collected 10 days post infection). Plots are faceted by age.

All rabbits treated with a high dose of RHDV2 IgG (independent of age group) survived virus challenge without showing obvious clinical signs in the 10 days following infection and had negligible amounts of viral RNA in the liver at post-mortem (Figure 3, Supplementary Figures S2 and S3, Supplementary Table S1). In contrast, all rabbits that did not receive RHDV2 IgG developed terminal RHD between 37 and 95 h ($\bar{x}$ = 51 h) post infection (hpi) and had very high viral RNA levels in the liver (Figure 3). These animals showed pyrexia and a reduction in activity levels as the disease progressed (Supplementary Figures S2 and S3). Of the animals treated with a low dose of RHDV2 IgG, all nine-week-old rabbits survived virus challenge, while 75% (3/4) 7 wo and 50% (2/4) five-week-old rabbits survived (Figure 3A). Animals that developed terminal disease had very high viral RNA loads in the liver, comparable to the ‘no antibody’ treatment groups, while low to moderate levels of viral RNA were detected in the livers of surviving rabbits (Figure 3B). The mortalities in the ‘low antibody’ treatment group ($\bar{x}$ = 121 hpi) occurred later than those in the ‘no antibody’ group ($\bar{x}$ = 53.2 hpi; *p* = 0.006, Welch two sample *t*-test), but characteristic clinical signs such as fever and lethargy were observed. A short episode of pyrexia and mild lethargy was observed in one of the three seven-week-old surviving rabbits in the ‘low antibody’ group; all other survivors remained healthy for the duration of the experiment (Supplementary Figures S2 and S3).

**Figure 4:**
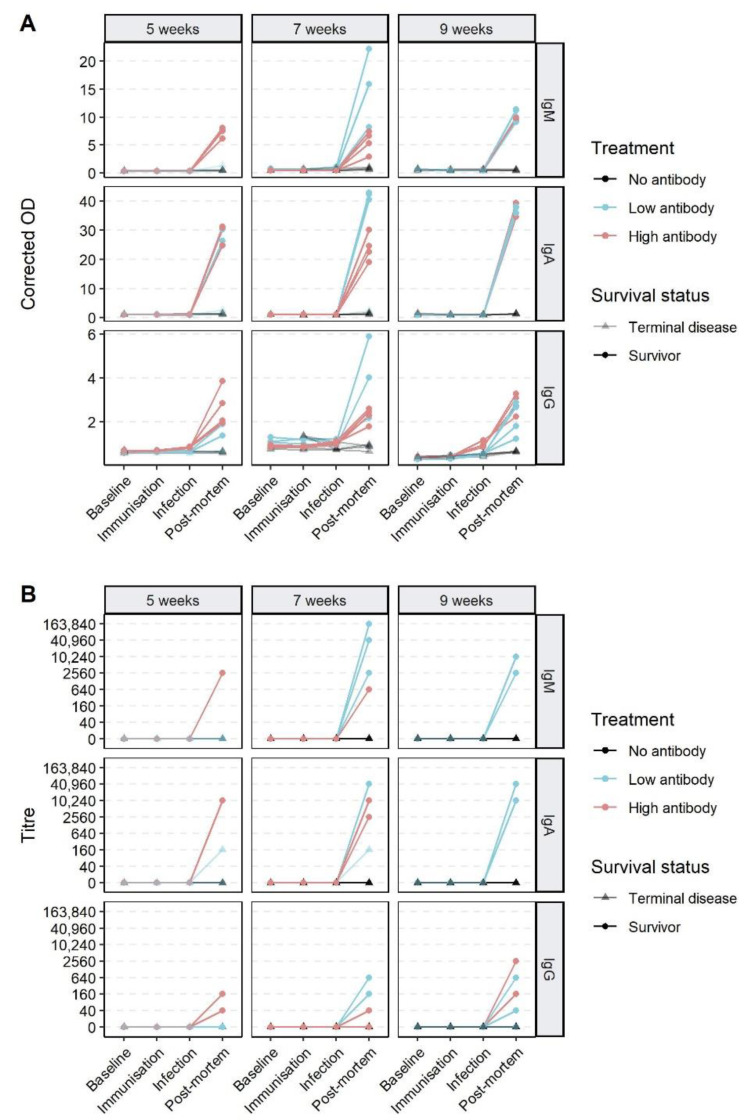
Serological responses of rabbits to RHDV2 passive immunisation and homologous virus challenge. Sera were collected at baseline, at the time of passive immunisation, at the time of infection (24 h after immunisation), and at post-mortem. Serological responses were evaluated with RHDV2 IgM, RHDV2 IgA, and RHDV IgG ELISAs. Results are expressed as the corrected optical density (OD) (the ratio of the OD of the sample to that of the negative control sera) (**A**), and as titres (**B**). Titres were calculated as the reciprocal of the highest serum dilution with an OD reading at least two times that of the negative control serum at the same dilution. Plots are faceted by age and serological assay.

To determine whether passively immunised animals were protected against disease or infection, we characterised the serological responses following RHDV2 IgG treatment and virus challenge (Figure 4). All surviving rabbits seroconverted after virus challenge, developing strong RHDV2 IgM and IgA responses. RHDV IgG titres at 10 dpi ranged from 0 to 2560, although even in animals with a titre of 0 an increase in corrected OD from baseline was detectable (Figure 4A). Two rabbits with terminal RHD (both ‘low dose’ treatment, one five-weeks-old and one seven-weeks-old wo) had IgA titres of 160 at post-mortem, but no IgM or IgG responses were detectable. Both these animals had prolonged survival times of 130 hpi.

While RHDV IgG antibody titres were not detectable 24 h after passive immunisation (i.e., at the time of infection), the ‘high dose’ group had significantly higher IgG corrected ODs than the controls in the seven-week-old and nine-week-old age groups (*p* = 0.015 and *p* = 0.002, respectively, Welch two sample *t*-test), reflective of the higher treatment dose.

## 4. Discussion

We hypothesised that passive immunisation against RHDV2, representative of maternal antibodies, would prevent both disease and infection after virus challenge. To test this, we passively immunised four 5-, 7-, and 9-week-old NZW rabbits with either low or high dose purified RHDV2 IgG. We orally challenged the rabbits with a highly virulent RHDV2 variant 24 h later. Although passive immunisation was not reliably detected by serology, all animals treated with a high dose of antibody and some of those treated with a low dose survived subsequent virus challenge. This confirms that passive immunity confers protection against lethal RHDV2 disease and that the level of immunity required for protection appears to be below the limit of detection, at least in the RHDV IgG ELISA. This concurs with previous findings for RHDV1 [14,16]. All surviving kittens developed a robust antibody response after virus challenge, demonstrating that the protection conferred by passive immunisation is not sterilising (i.e., attenuating). This differs from the findings of Robinson et al. [16] for RHDV1, who reported that, while most kittens from immune does seroconverted after virus challenge, some with higher maternal antibody levels developed neutralising responses to infection. It is unclear if these discrepancies are due to different experimental protocols (e.g., IM vs. oral route of challenge), or if our passive immunisation protocol did not result in titres sufficient to achieve neutralisation. Additional work would be required to test if higher doses of RHDV2 IgG may protect against RHDV2 oral infection.

While there was a trend towards increased mortality in younger animals after low dose passive immunisation, our study was insufficiently powered to assess the effects of age with confidence. If substantiated, this could be directly related to physiological changes associated with age, or it is possible that this is an artefact of the weight-based dosing regimen used here [47]. Potentially, a surface area-based dosing regimen would be more appropriate for RHDV2 IgG.

In the context of rabbit biocontrol, these findings suggest that young rabbits with maternal antibodies will not only survive the RHDV2 challenge but will also seroconvert, leading to long-term protection against re-infection. Indeed, even low doses of RHDV2 IgG appear to be protective; therefore, a negative result in the RHDV IgG ELISA may not indicate susceptibility, as has previously been reported for RHDV1 [16]. If RHDV2 was approved as a biological control, any release would need to account for the presence of maternal antibodies in the target population. Clearly, the development of new serological assays that can more sensitively detect meaningful antibody levels in individuals are warranted, and more research is needed to understand the correlates of immunity to RHDV2.

In the context of endemic circulation of the virus, RHDV2 appears to enter populations as soon as new susceptible animals are present [48]. This susceptible cohort is likely to be young rabbits, and therefore maternal antibodies are likely to be present. The results from our study suggest that this would rapidly lead to an increase in RHDV2 seropositivity in the population, since exposed, maternally protected kittens will likely survive infection and seroconvert. Interestingly, rabbits of less than two-to-four weeks old are typically unable to mount normal specific immune responses to antigens [30]. Therefore, if RHDV2 enters a population with a large proportion of very young kittens, then high mortality may still be observed. Additional studies would be required to verify this experimentally.

## Figures and Tables

**Table 1:** Characteristics of the different ELISAs used in this study.

Assay	Sensitivity	Cross-Reactivity with Other Lagoviruses	Reference
RHDV2 IgM	High	Moderate	[35]
RHDV2 IgA	High	Moderate	[35]
RHDV IgG	High	High	[33]

## Data Availability

All supporting data are included in this manuscript.

## Supplementary Materials

The following are available online at www.mdpi.com/article/10.3390/vaccines9101197/s1, Figure S1: Reducing SDS-PAGE of purified RHDV2 IgG preparations, Figure S2: Temperature changes in passively immunised rabbits following RHDV2 infection, Figure S3: Activity changes in passively immunised rabbits following RHDV2 infection. Table S1: Daily mean clinical scores following virus challenge.
