# Supplementary material for: Passive Immunisation against RHDV2 Induces Protection against Disease but Not Infection
Source: Vaccines (Basel). 2021 Oct 18;9(10):1197. doi: 10.3390/vaccines9101197 (PMC8537872; doi:10.3390/vaccines9101197)
Supplement: Supplementary file 1 [file vaccines-09-01197-s001.zip › vaccines-1405114-supplementary.pdf]

Supplementary materials

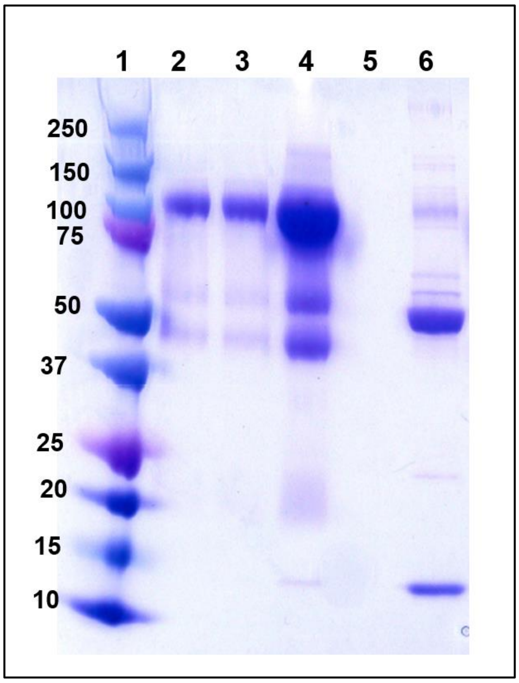

Supplementary Figure S1. Reducing SDS-PAGE of purified RHDV2 IgG preparations. RHDV2 IgG was purified by FPLC from hyperimmune rabbit serum (lane 6), dialysed against PBS (lane 2), filter sterilised ('low dose preparation'; lane 3), and concentrated ('high dose preparation'; lane 4). Samples were run on a TruPage Precast gel 4-20% (Sigma-Aldrich) in TruPage TEA-Tricine SDS running buffer at 180 V for 45 minutes and were stained with Coomassie R250 overnight followed by destaining for 4.5 hours. Lane 1 is a molecular weight marker (Bio-Rad #1610374). Lane 5 shows the flow-through from the concentration step. Hyperimmune serum in lane 6 was diluted 0.1x in PBS. Equal volumes of each preparation were added to lanes 2–6.

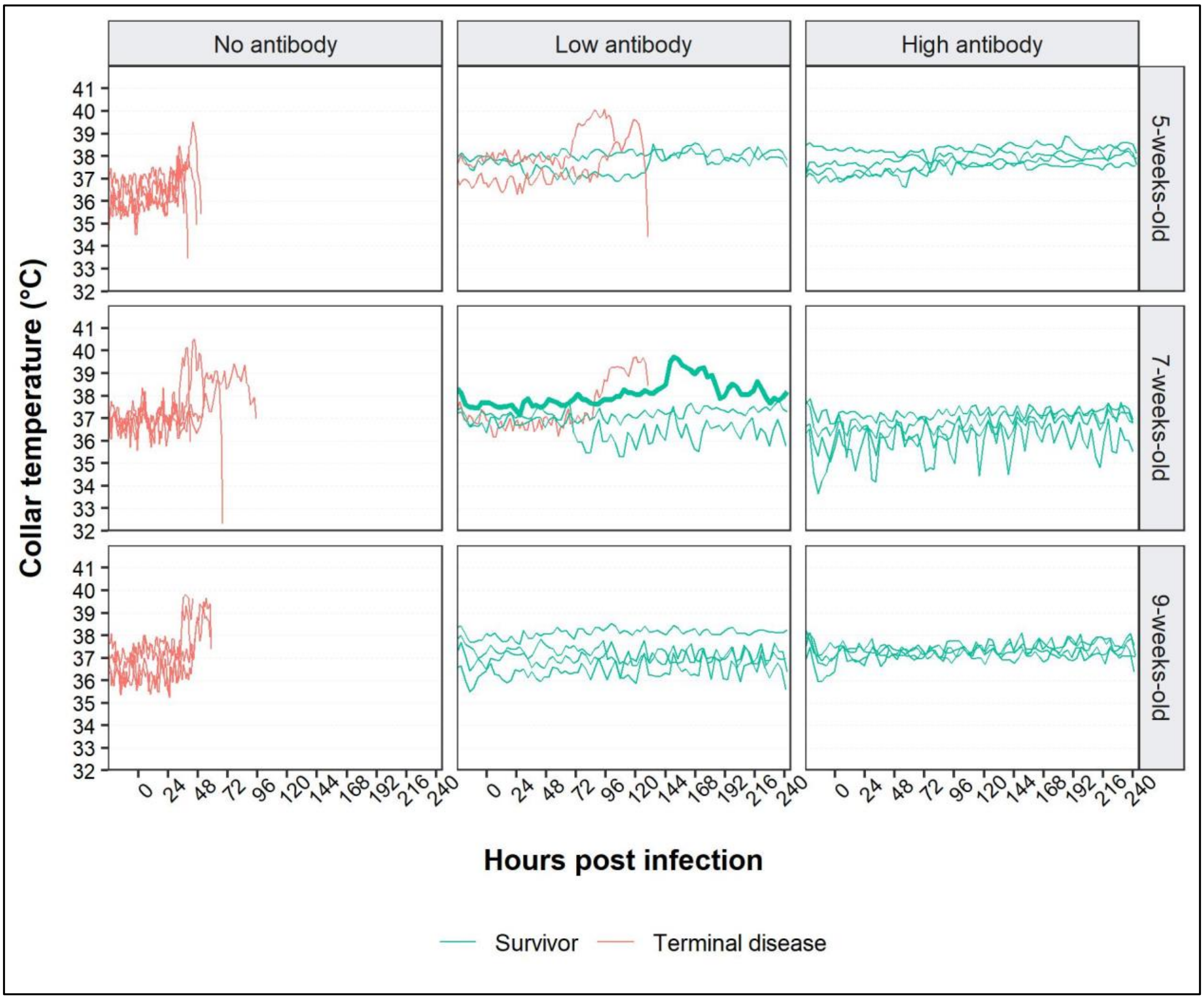

Supplementary Figure S2. Temperature changes in passively immunised rabbits following RHDV2 infection. Each animal was fitted with a collar containing a Sub-Cue Mini temperature datalogger to measure external body temperature. Temperature was plotted from the time of antibody injection (24 hours prior to infection) to 10 days post infection. Plots are faceted by age and treatment group. Each line represents the smoothed conditional mean temperature for an individual rabbit. This was calculated using local polynomial regression fitting as implemented in ggplot2 (geom_smooth(method=”loess”) with span = 0.05). Lines are coloured by survival status. The thick line represents K629, which developed pyrexia but recovered.

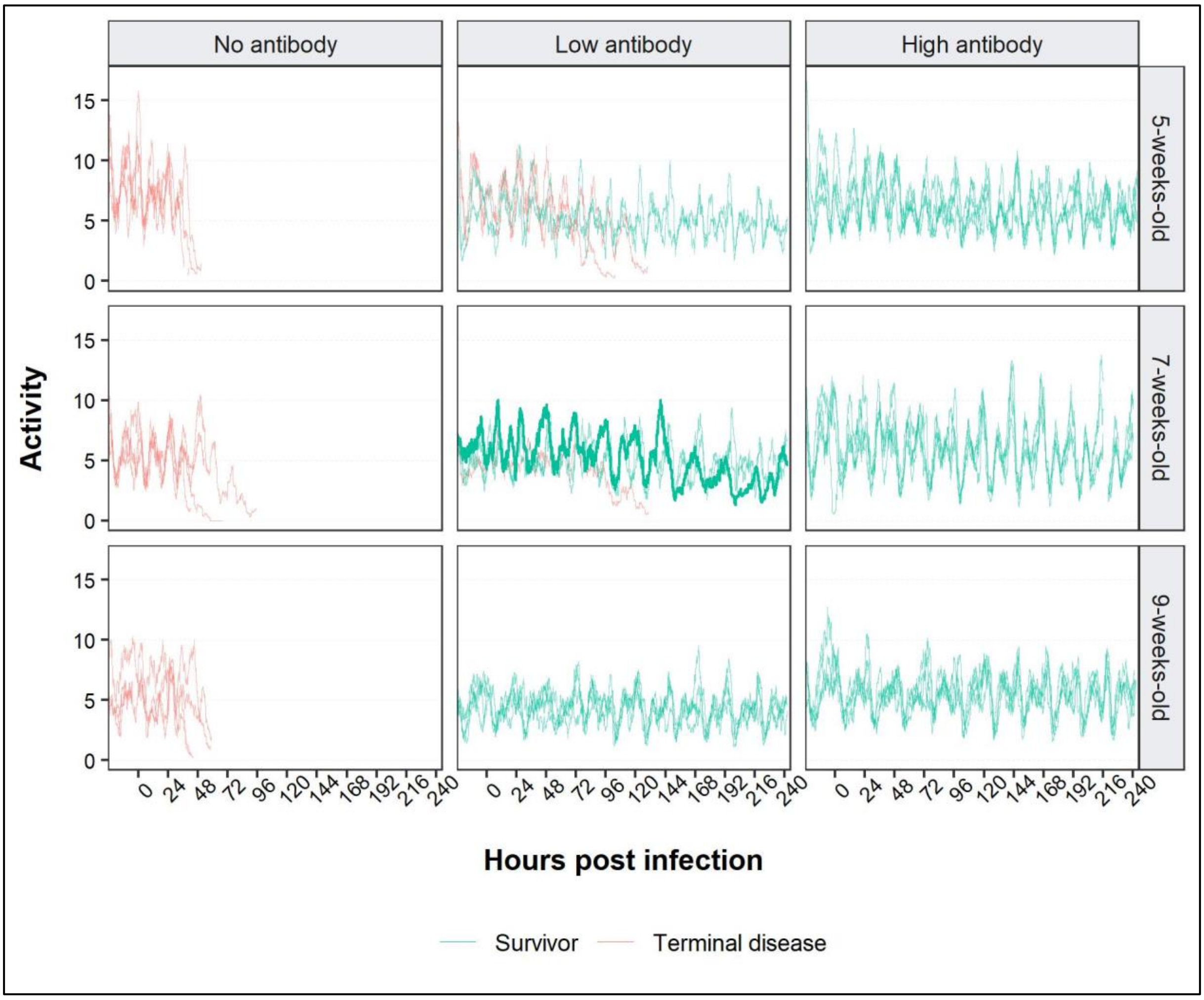

Supplementary Figure S3. Activity changes in passively immunised rabbits following RHDV2 infection. Each animal was fitted with a collar containing a FitBark2 activity monitor. This measures motion in arbitrary units through a 3D-accelerometer. Activity was plotted from the time of antibody injection (24 hours prior to infection) to 10 days post infection. Plots are faceted by age and treatment group. Each line represents the simple moving average of activity levels for an individual rabbit. This was calculated using the movavg function as implemented in the pracma package with a backwards window length of 255. Lines are coloured by survival status. The thick line represents K629, which developed pyrexia but recovered.

Supplementary Table S1. Daily mean clinical scores following virus challenge. Observations of body weight[*], rectal temperature[†], feed intake[‡], urine and faecal output[§], behaviour[‖], and reactivity to handling[¶] were recorded twice daily. A daily mean clinical score (ranging from 0–1) was calculated by averaging the observations for all parameters across both observation points each day. Note that many of these clinical signs are not specific to rabbit haemorrhagic disease and can also be observed with other pathologies or with stress.

			Daily mean clinical score										
**Animal ID**	**Age**	**Treatment**	**Day 0**	**Day 1**	**Day 2**	**Day 3**	**Day 4**	**Day 5**	**Day 6**	**Day 7**	**Day 8**	**Day 9**	**Day 10**
K603	5 weeks	No antibody	0.09	0.50									
K604	5 weeks	No antibody	0.00	0.30	1.00								
K606	5 weeks	No antibody	0.00	0.30	0.83								
K612	5 weeks	No antibody	0.09	0.10	0.67								
K666	5 weeks	Low antibody	0.09	0.00	0.00	0.00	0.00	0.00	0.09	0.00	0.00	0.00	0.00
K667	5 weeks	Low antibody	0.00	0.00	0.00	0.00	0.00	0.09	0.09	0.00	0.00	0.00	0.17
K674	5 weeks	Low antibody	0.00	0.00	0.00	0.27	0.91						
K681	5 weeks	Low antibody	0.00	0.00	0.00	0.00	0.18	0.57					
K673	5 weeks	High antibody	0.00	0.00	0.00	0.09	0.00	0.00	0.00	0.00	0.00	0.00	0.00
K675	5 weeks	High antibody	0.00	0.00	0.09	0.00	0.09	0.00	0.00	0.00	0.00	0.00	0.00
K679	5 weeks	High antibody	0.00	0.00	0.00	0.00	0.00	0.00	0.00	0.09	0.09	0.09	0.00
K680	5 weeks	High antibody	0.09	0.00	0.00	0.00	0.00	0.00	0.00	0.09	0.00	0.00	0.00
K619	7 weeks	No antibody	0.09	0.00	0.64								
K627	7 weeks	No antibody	0.09	0.09	0.27	1.00							
K632	7 weeks	No antibody	0.00	0.00	0.18	0.73	0.50						
K686	7 weeks	No antibody	0.00	0.00	0.50								
K629	7 weeks	Low antibody	0.00	0.09	0.09	0.00	0.00	0.09	0.27	0.27	0.00	0.09	0.00
K618	7 weeks	Low antibody	0.00	0.00	0.18	0.18	0.36	0.86					
K624	7 weeks	Low antibody	0.09	0.00	0.09	0.00	0.00	0.09	0.00	0.09	0.09	0.00	0.00
K625	7 weeks	Low antibody	0.00	0.00	0.09	0.09	0.00	0.09	0.09	0.09	0.00	0.00	0.00
K685	7 weeks	High antibody	0.09	0.00	0.09	0.00	0.00	0.00	0.20	0.00	0.09	0.00	0.00
K687	7 weeks	High antibody	0.18	0.09	0.09	0.18	0.09	0.11	0.10	0.09	0.00	0.00	0.00
K688	7 weeks	High antibody	0.09	0.09	0.09	0.00	0.00	0.00	0.10	0.00	0.00	0.00	0.00
K689	7 weeks	High antibody	0.09	0.09	0.27	0.09	0.00	0.11	0.20	0.09	0.00	0.00	0.00
K648	9 weeks	No antibody	0.00	0.18	1.00								
K651	9 weeks	No antibody	0.09	0.18	1.00								
K652	9 weeks	No antibody	0.09	0.00	0.83								
K657	9 weeks	No antibody	0.00	0.09	0.64								
K650	9 weeks	Low antibody	0.18	0.18	0.27	0.09	0.00	0.00	0.00	0.00	0.00	0.13	0.17
K653	9 weeks	Low antibody	0.09	0.09	0.18	0.09	0.00	0.13	0.00	0.00	0.00	0.13	0.00

K654	9 weeks	Low antibody	0.27	0.18	0.27	0.09	0.00	0.13	0.00	0.00	0.00	0.00	0.00
K656	9 weeks	Low antibody	0.09	0.09	0.09	0.00	0.00	0.00	0.00	0.00	0.00	0.00	0.00
K640	9 weeks	High antibody	0.18	0.18	0.09	0.09	0.10	0.00	0.00	0.00	0.00	0.00	0.00
K641	9 weeks	High antibody	0.09	0.09	0.00	0.09	0.00	0.00	0.00	0.00	0.00	0.00	0.17
K649	9 weeks	High antibody	0.18	0.18	0.09	0.00	0.00	0.00	0.00	0.00	0.00	0.00	0.00
K655	9 weeks	High antibody	0.00	0.09	0.18	0.00	0.00	0.00	0.00	0.00	0.00	0.00	0.00

* 0 weight gain, 1 no weight gain

† 0 rectal temperature <40°C, 1 rectal temperature >40°C

‡ 0 feed intake >50 g per day, 1 feed intake ≤ 50 g per day

§ 0 urine and faecal output normal, 1 urine and faecal output reduced

‖ 0 behaviour normal, 1 lethargy or other abnormal behaviour

¶ 0 normal muscle tone and reactivity on handling, 1 weakness and/or minimal reactivity on handling
